# Transcriptome Analysis of Circulating Immune Cell Subsets Highlight the Role of Monocytes in Zaire Ebola Virus Makona Pathogenesis

**DOI:** 10.3389/fimmu.2017.01372

**Published:** 2017-10-26

**Authors:** Andrea R. Menicucci, Krista Versteeg, Courtney Woolsey, Chad E. Mire, Joan B. Geisbert, Robert W. Cross, Krystle N. Agans, Allen Jankeel, Thomas W. Geisbert, Ilhem Messaoudi

**Affiliations:** ^1^Division of Biomedical Sciences, University of California, Riverside, Riverside, CA, United States; ^2^Galveston National Laboratory, Galveston, TX, United States; ^3^Department of Microbiology and Immunology, University of Texas Medical Branch, Galveston, TX, United States; ^4^Department of Molecular Biology and Biochemistry, College of Biological Sciences, University of California, Irvine, Irvine, CA, United States

**Keywords:** Ebola, hemorrhagic fever, pathogenesis, RNASeq, monocytes

## Abstract

Existing models of Ebola virus disease (EVD) suggest antigen-presenting cells are initial targets of *Zaire ebolavirus* (ZEBOV). *In vitro* studies have shown that ZEBOV infection of monocytes and macrophages results in the production of inflammatory mediators, which may cause lymphocyte apoptosis. However, these findings have not been corroborated by *in vivo* studies. In this study, we report the first longitudinal analysis of transcriptional changes in purified monocytes, T-cells, and B-cells isolated from cynomolgus macaques following infection with ZEBOV-Makona. Our data reveal monocytes as one of the major immune cell subsets that supports ZEBOV replication *in vivo*. In addition, we report a marked increase in the transcription of genes involved in inflammation, coagulation, and vascular disease within monocytes, suggesting that monocytes contribute to EVD manifestations. Further, genes important for antigen presentation and regulation of immunity were downregulated, potentially subverting development of adaptive immunity. In contrast, lymphocytes, which do not support ZEBOV replication, showed transcriptional changes limited to a small number of interferon-stimulated genes (ISGs) and a failure to upregulate genes associated with an antiviral effector immune response. Collectively, these data suggest that ZEBOV-infected monocytes play a significant role in ZEBOV-Makona pathogenesis and strategies to suppress virus replication or modify innate responses to infection in these cells should be a priority for therapeutic intervention.

## Introduction

*Ebolaviruses* (EBOV) are among the deadliest re-emerging viruses; causing hemorrhagic fever with case fatality rates (CFRs) ranging from 40 to 90% depending on the species. Ebola virus disease (EVD) is characterized by an excessive inflammatory response, lymphocyte apoptosis, vascular impairment, hemorrhage, and coagulation defects leading to multi-organ failure and shock (1, 2). *Zaire ebolavirus* (ZEBOV) is responsible for the highest CFRs and most outbreaks, including the largest epidemic that originated December 2013 in West Africa. The ZEBOV variant responsible for this epidemic, Makona, affected over 28,600 individuals and claimed over 11,300 lives (3).

Despite its impact on human health, the molecular basis for EVD is incompletely understood. Much of what is known about ZEBOV pathogenesis has been acquired through infectious studies in nonhuman primates (NHPs), particularly cynomolgus and rhesus macaques. Studies in macaques have shown that monocytes, macrophages, and dendritic cells (DCs) are the initial targets of ZEBOV (4). Our understanding of how ZEBOV impacts function and behavior of DC and monocytes/macrophages has been primarily extrapolated from *in vitro* infections. Data from these studies show that *in vitro* infection of monocytes and macrophages with ZEBOV triggers a robust expression of inflammatory mediators including IL-1β, IL-6, IL-8, MIP-1α, MIP-1β, MCP-1, and TNFα (5–7), several of which have been detected in the plasma of humans and animal models following ZEBOV infection (5, 8–12). Inflammatory mediators released by monocytes may also contribute to the impairment of the vascular system and disseminated intravascular coagulation as well as lymphocyte death (13–16). However, whether monocytes are the major contributors to inflammation following ZEBOV infection *in vivo* remains to be elucidated. Moreover, the susceptibility of monocytes to ZEBOV remains contradictory. Some *in vitro* studies reported successful ZEBOV replication in both primary monocytes and macrophages (6, 7, 15); others indicated ZEBOV entry is delayed in primary compared to differentiated monocytes and THP-1 cells are refractory to entry until PMA-induced differentiation (17).

Severe lymphopenia is a hallmark of ZEBOV infection (4, 12, 18, 19), observed as a loss of peripheral blood CD4^+^ and CD8^+^ T-cells, as well as natural killer cells in cynomolgous macaques (20, 21) and humans (22). The loss of B-cells has been controversial with some NHP studies reporting apoptosis of B lymphocytes (15), while others observe no changes in B-cell counts (20). *In vivo* and *in vitro* studies using TUNEL staining and transmission electron microscopy confirm apoptosis as the main mechanism of lymphocyte loss during ZEBOV infection (14, 15). Furthermore, while analysis of ZEBOV-infected NHP tissues shows the presence of ZEBOV antigens within phagocytic cells 3–4 days post challenge, no ZEBOV antigens have been observed in T- and B-cells throughout infection. This suggests lymphocyte apoptosis during ZEBOV infection is not due to direct viral replication but rather inflammatory mediators, such as TNFα, nitric oxide, and reactive oxygen species. Presumably these cytokines and chemokines are produced in response to infection of phagocytes and other cells as well as immunosuppressive EBOV peptides (15, 23–25). However, the effects of ZEBOV infection and these mediators on T- and B-cell function remains incompletely defined.

Although many genomic studies have provided us with insight into the global transcriptional changes as disease progresses, studies that elucidate the role of individual immune cell subsets in viral pathogenesis are lacking. In this study, we used RNA sequencing (RNA-Seq) to uncover longitudinal gene expression profiles within monocytes, T-cells, and B-cells purified from ZEBOV-Makona infected cynomolgus macaque peripheral blood mononuclear cells (PBMC) at different times post infection. Our data identify monocytes as one of the major targets of infection *in vivo*. Moreover, gene expression profiles at later stages of disease strongly support the role of monocytes as a significant source of inflammatory cytokines and chemokines during ZEBOV infection. In contrast, lymphocytes exhibited limited transcriptional activity and lacked significant signs of an effective adaptive immune response.

## Materials and Methods

### Study Design

This study was designed to elucidate the role of specific immune cell subsets in mediating ZEBOV-Makona pathogenesis. For this purpose, monocytes, T-cells, and B-cells were isolated by magnetic bead separation from PBMC collected on days 0 (*n* = 10), 1 (*n* = 5), 2 (*n* = 4), 3 (*n* = 3), 4 (*n* = 2), 5 (*n* = 2), and 6 (*n* = 4) post ZEBOV-Makona infection from cynomolgus macaques (26). Challenges with allowable volumes of blood collection and cell separation within a Biosafety Level 4 (BSL-4) lab precluded us from having *n* > 2 for days 4 and 5 post infection. RNA-Seq was used to measure host gene expression changes within each cell subset.

### Virus and Challenge

A laboratory seed stock of the Makona variant isolate H.sapiens-tc/GIN/2014/Makona-Gueckedou-C07?(accession number KJ660347.2) was grown from the serum of a 2014 fatal human case in Guékédou, Guinea and passaged twice in authenticated Vero E6 cells (ATCC, CRL-1586). This clone was selected because it is one of the earliest and well characterized isolates from the EBOV epidemic in West Africa that was also used in a recent NHP study (27). Ten healthy, filovirus-negative male cynomolgus macaques (*Macaca fascicularis*) 3–5 years of age and between 4 and 8 kg were challenged with 1,000 pfu of ZEBOV-Makona intramuscularly with the dose divided equally into the left and right quadriceps. Animals were housed in the BSL-4 laboratory in the Galveston National Laboratory and monitored post challenge for clinical signs of disease including temperature, weight loss, behavioral changes, changes in blood count, and blood chemistries.

### Sample Collection and PBMC Isolation

Blood was collected by venipuncture into EDTA tubes. To isolate PBMC, whole blood was centrifuged over Histopaque (Sigma-Aldrich, St. Louis, MO, USA) using AccuSpin Tubes (Sigma-Aldrich, St. Louis, MO, USA) at ~400 × *g* for 45 min, room temperature with no brake. PBMC were counted on a TC20 Automated Cell Counter (Bio-Rad, Hercules, CA, USA).

### Magnetic Bead Cell Separation

Peripheral blood mononuclear cells underwent sequential separations using magnetic microbeads (Miltenyi Biotec, San Diego, CA, USA) as described in Figure S1 in Supplementary Material. PBMC were initially stained with anti-CD2 microbeads to isolate T and NK cells. The CD2 negative cell population was then stained with anti-CD20 microbeads to isolate the B-cell population. The CD20 negative fraction was collected and stained with CD14 microbeads to isolate monocytes. Purity of the fractions was confirmed using flow cytometry (Table S1 in Supplementary Material). All samples were acquired using a BD FACS Canto-II (Becton Dickinson Biosciences, San Jose, CA, USA) using BD FACS Diva software. Live cells were identified by FSC and SSC and a minimum of 50,000 events were collected for each sample. Data was analyzed using FlowJo Analysis Software (FlowJo LLC, Ashland, OR, USA) and Prism Software (GraphPad Software, Irvine, CA, USA). Only samples with purity above the cutoff of 60% were included in the analysis. The number of samples available at each time point were as follows: monocyte samples on days 0 (*n* = 5), 1 (*n* = 4), 2 (*n* = 4), 3 (*n* = 2), 4 (*n* = 2), and 6 (*n* = 3) post infection; T-cell samples on days 0 (*n* = 7), 1 (*n* = 4), 2 (*n* = 4), 3 (*n* = 2), 4 (*n* = 2), 5 (*n* = 2), and 6 (*n* = 3) post infection; and B-cell samples on days 0 (*n* = 2), 1 (*n* = 4), 2 (*n* = 4), and 6 (*n* = 4) post infection. Unfortunately, a sufficiently pure DC population could not be obtained; therefore, in this study, we focused on monocytes, T-cells, and B-cells.

### Library Preparation for RNA-Seq

RNA from each cell subset was isolated using Zymo Research Direct-zol RNA mini-prep (Zymo Research, Irvine, CA, USA) per manufacturer’s instructions. RNA concentration and integrity was determined using an Agilent 2100 Bioanalyzer (Agilent Technologies, Santa Clara, CA, USA). Ribosomal RNA (rRNA) was depleted using the ClontechRibo-Gone rRNA Removal kit. Libraries were constructed using the ClontechSMARTer Stranded RNA-Seq kit (Takara Bio Inc., Kusatsu, Shiga, Japan). First, rRNA-depleted RNA was fragmented and converted to double stranded cDNA. Adapters were ligated and the ~300 base pair long fragments were then amplified by PCR and selected by size exclusion. Each library was prepared with a unique indexed primer for multiplexing. In order to ensure proper sizing, quantitation, and quality prior to sequencing, libraries were analyzed on the Agilent 2100 Bioanalyzer. Multiplexed libraries were subjected to single-end 100 base pair sequencing using the Illumina HiSeq2500 platform (Illumina, San Diego, CA, USA).

### Bioinformatic and Statistical Analysis

Data analysis was performed with the RNA-Seq workflow module of the systemPipeR package available on Bioconductor (28). RNA-Seq reads were demultiplexed, quality filtered, and trimmed using Trim Galore with an average phred score cutoff of 30 and minimum length of 75bp. Three base pairs from the 5′ end were trimmed as per Clontech’s instruction. Quality reports were generated with the FastQC function. Because the complete genome annotation for *M. fascicularis* is not available, the *Macaca mulatta* genome sequence (Macaca_mulatta.MMUL_1.dna.toplevel.fa) and annotation file from Ensembl (Macaca_mulatta.MMUL_1.78.gtf) was used. In order to determine the level of viral transcription at different time points, the ZEBOV variant Makona genome (H.sapiens-wt/GIN/2014/Makona-Gueckedou-C07) from Virus Pathogen Resource was adjoined to the *M. mulatta* reference. ZEBOV open reading frames (ORFs), intergenic regions (IGRs), and leader and trailing sequences were defined based on the ZEBOV-Makona genome annotation GTF file: NP (470–2689), VP35 (3129–4151), VP40 (4479–5459), GP (6039–8068), VP30 (8509–9375), VP24 (10345–11100), L (11581–18219), Leader (1–469), IGR_NP_VP35 (2690–3128), IGR_VP35_VP40 (4152–4478), IGR_VP40_GP (5460–6038), IGR_GP_VP30 (8069–8508), IGR_VP30_VP24 (9376–10344), IGR_VP24_L (11101–11580), and Trailing (18220–18959). RNA-Seq reads were mapped with the alignment suite Bowtie2/Tophat2 against a reference genome containing both *M. mulatta* and ZEBOV-Makona genome sequences. Raw expression values in the form of gene-level read counts were generated with the *summarizeOverlaps* function, counting only the reads overlapping exonic regions of genes, and discarding reads mapping to ambiguous regions of exons from overlapping genes. Normalization and statistical analysis of differentially expressed genes (DEGs) was performed using the *edgeR* package, which normalizes reads by the trimmed mean of M values method. RNA-seq data presented in this article were submitted to the National Center for Biotechnology Information Sequence Read Archive (BioProject accession PRJNA412109). DEGs were defined as those with a fold change ≥2 and a false discovery rate (FDR) corrected *p*-value ≤ 0.05 compared to 0 days post-infection (DPI). Only protein coding genes with human homologs and an average 5 reads per kilobase of transcript per million mapped reads (RPKM) were included for further analysis. Reads mapping to the ZEBOV-Makona genome were normalized as RPKM. Heatmaps, venn diagrams, and a violin plot were generated using R packages gplot, DESeq2, VennDiagram, and ggplot2. Heatmaps for gene expression represent the absolute normalized expression (RPKM); range of colors is based on scaled and centered RPKM values of the entire set of genes (red represents increased expression while blue represents decreased expression). Heatmaps for functional enrichment of DEGs represent –log_10_(FDR-corrected p-value); range of colors is based on the lowest and highest –log_10_(FDR) values for the entire set of terms; the number of DEGs mapping to each functional enrichment term each day is listed within each box; blank boxes represent no statistical significance.

Differentially expressed genes detected in monocytes 6 DPI were compared to the transcriptional profile of monocyte-derived macrophages (MDMs) and macrophages differentiated from human induced pluripotent stem cells following 6-h lipopolysaccharide (LPS) stimulation (29) using R package VennDiagram.

### Functional Enrichment

Functional enrichment of DEGs was done to identify clusters of genes mapping to specific biological pathways, specifically Gene Ontology (GO) terms and Diseases (by Biomarkers) using MetaCore™ (Thomson Reuters, New York, NY, USA). Significant functional enrichment terms were defined as those with a FDR corrected *p*-value ≤ 0.05. Since this software requires human gene identifiers for analysis, rhesus DEGs were mapped to human homologs using BioMart.

### Statistical Analysis

Statistical analysis of viral genome copy number; hematology and clinical chemistry data; cytokine, chemokine, and growth factor data; and flow cytometry data were carried out using the SAS software, PROC MIXED (Figure S2 in Supplementary Material; Table 1). A repeated measures analysis was used to model each of the dependent variables. Intra-animal correlation was modeled using a compound symmetric variance-covariance structure. In some cases, a linear trend was used for the mean. In other cases, in which a linear trend was not a good fit, a nonparametric trend was used where each time point was modeled by its own mean. Missing data were handled by using maximum likelihood algorithms to fit the model. When a linear model was an adequate trend, the *p*-value for the estimated slope was reported. When each time point was modeled by its own mean, the mean response at each non-zero time point was contrasted with the mean response at the zero time point. Holm’s multiple comparison method was used to adjust the *p*-values for each contrast (30).

**Table 1:** Summary of average clinical data and flow cytometry data during ZEBOV-Makona infection.

Days post infection	1	2	3	4	5	6
Number of samples	10	10	6	6	4	4
Viral load (genome copy/mL)	50	50	1.89E06	3.12E08	1.37E09	3.68E10
Body temperature (°F)	101.1	100.5	100.8	103.5	104.95	104.2
Rash (fraction of animals)					2/4	3/4
Anorexia, depression (fraction of animals)						4/4
Weakness (fraction of animals)						2/4
Hemorrhage (fraction of animals)						1/4
AST levels (U/L) (average ± SEM)	51 ± 0.04	64.3 ± 0.03	42 ± 0.03	54.5 ± 0.04	71 ± 0.06	278.75* ± 0.07
CRP levels (mg/L) (average ± SEM)	7.2 ± 0.05	4 ± 0.07	4.1 ± 0.08	25.3* ± 0.07	112.1* ± 0.07	116.5* ± 0.11
BUN levels (mg/dL) (average ± SEM)	20 ± 1.53	26.5* ± 1.42	14.7 ± 1.48	13 ± 1.70	16 ± 2.04	23.5* ± 2.44
Platelet levels (×10^3^/μL) (average ± SEM)	357 ± 0.03	375.8 ± 0.04	342 ± 0.04	310.5 ± 0.04	205* ± 0.04	162* ± 0.04

*p-value ≤ 0.05 compared to day 0; average ± standard error of predicted mean is shown.

## Results

### Disease Course Following Makona Infection

**Figure 1:**
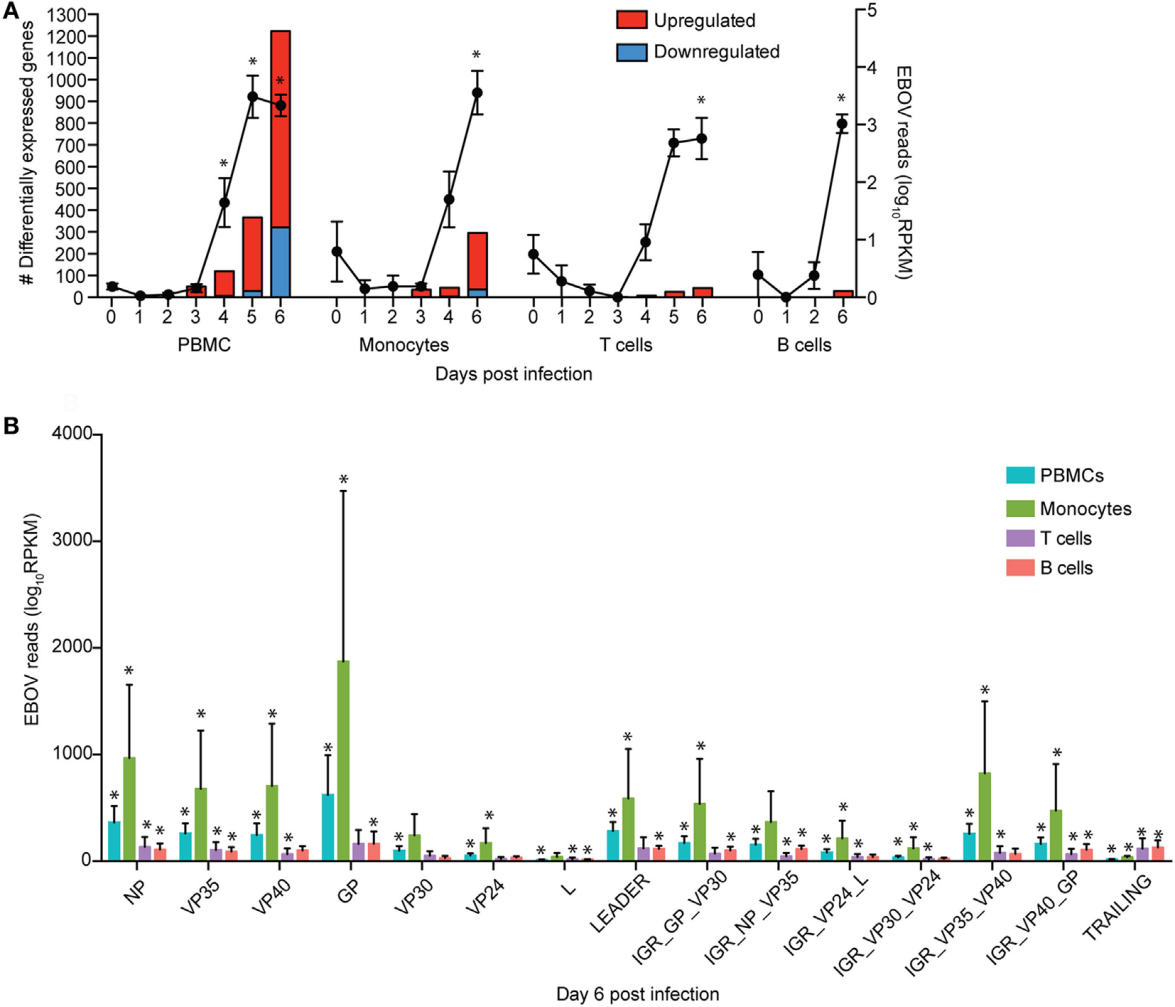
Monocytes support ZEBOV-Makona replication and are major contributors of inflammation. **(A)** Bar graph depicts number of protein coding differentially expressed genes (DEGs; defined as those ≥2 fold change and false discovery rate (FDR) corrected *p*-value ≤ 0.05 compared to 0 DPI) that have human homologs (red indicates upregulated while blue indicates downregulated DEGs) in peripheral blood mononuclear cells (PBMC), monocytes, T cells, and B cells. Line graph represents number of normalized viral transcripts (RPKM); mean ± SEM are shown; * *p* ≤ 0.05 compared to day 0. **(B)** RPKM normalized RNA sequencing reads mapping to each ZEBOV open reading frame (ORF), intergenic region (IGR), as well as leader and trailing sequences 6 DPI within PBMC, monocytes, T-cells, and B-cells. FDR adjusted *p*-value was obtained following *EdgeR* analysis; mean ± SEM are shown; *p ≤ 0.05 compared to day 0.

Viremia was first detected 3 DPI and increased as disease progressed (Table 1). Clinical signs (fever, rash, bleeding), liver and kidney dysfunction, decreased platelet count, and increased inflammatory cytokine and chemokine levels were evident 5–6 DPI (Table 1; Figure S2A in Supplementary Material). Although the number of circulating monocytes, DCs, and NK cells remained relatively stable (Figure S2B in Supplementary Material), we observed a transient increase in the number of activated monocytes 4 DPI (Figure S2B in Supplementary Material). In contrast, the number of CD4^+^ T-cells, CD8^+^ T-cells, and B-cells as well as their naïve and memory subsets significantly decreased as infection progressed (Figure S2B in Supplementary Material). Transcriptional analysis of PBMC following infection shows the number of DEGs correlated with viral loads, with the largest changes detected 6 DPI (Figure 1A). Functional enrichment revealed that DEGs upregulated 6 DPI play a role in innate immunity, inflammation, and cell death, while downregulated DEGs are important for cell motility, signaling, and adaptive immunity (Table 2). These findings are described in greater detail in our earlier study (26).

**Table 2:** Functional enrichment of differentially expressed genes (DEGs) in peripheral blood mononuclear cells 6 DPI.

Gene Ontology	False discovery rate adjusted *p*-value	Number of DEGs
**Upregulated**		
Immune system process	3.02^−82^	358
Defense response	2.19^−63^	255
Response to stress	2.73^−61^	418
Innate immune response	7.57^−47^	154
Response to cytokine	1.34^−45^	173
Inflammatory response	1.61^−34^	117
Cell death	7.53^−32^	158
Signal transduction	9.15^−29^	436
Positive regulation of metabolic process	1.07^−28^	312
Myeloid leukocyte activation	3.05^−23^	47
**Downregulated**		
Regulation of signal transduction	6.16^−10^	103
Leukocyte activation	8.85^−10^	40
Immune system process	1.51^−9^	90
Lymphocyte activation	1.08^−8^	34
Regulation of cell motility	6.82^−11^	44
Positive regulation of metabolic process	1.06^−7^	104
Adaptive immune response	9.93^−7^	25
Regulation of blood coagulation	2.49^−6^	13

### Monocytes Support ZEBOV-Makona Replication and Are Major Contributors of Inflammation

To investigate the impact of ZEBOV-Makona infection within monocytes, we determined longitudinal changes in gene expression profiles using RNA-Seq. The average purity of monocytes analyzed at the various time points was >70% (Table S1 in Supplementary Material). Transcriptional changes correlated with ZEBOV reads (Figure 1A). We further investigated the total number of viral reads mapping to each ZEBOV ORF and IGR within purified monocytes (Figure 1B). At 6 DPI, viral transcripts were abundant with ORF L and the trailing end region having the lowest average RPKM values, while ORFs GP and NP had the highest. Based on Euclidean distances, the viral transcript profile in monocytes was most comparable to that observed in PBMC (Figure S3 in Supplementary Material).

**Figure 2:**
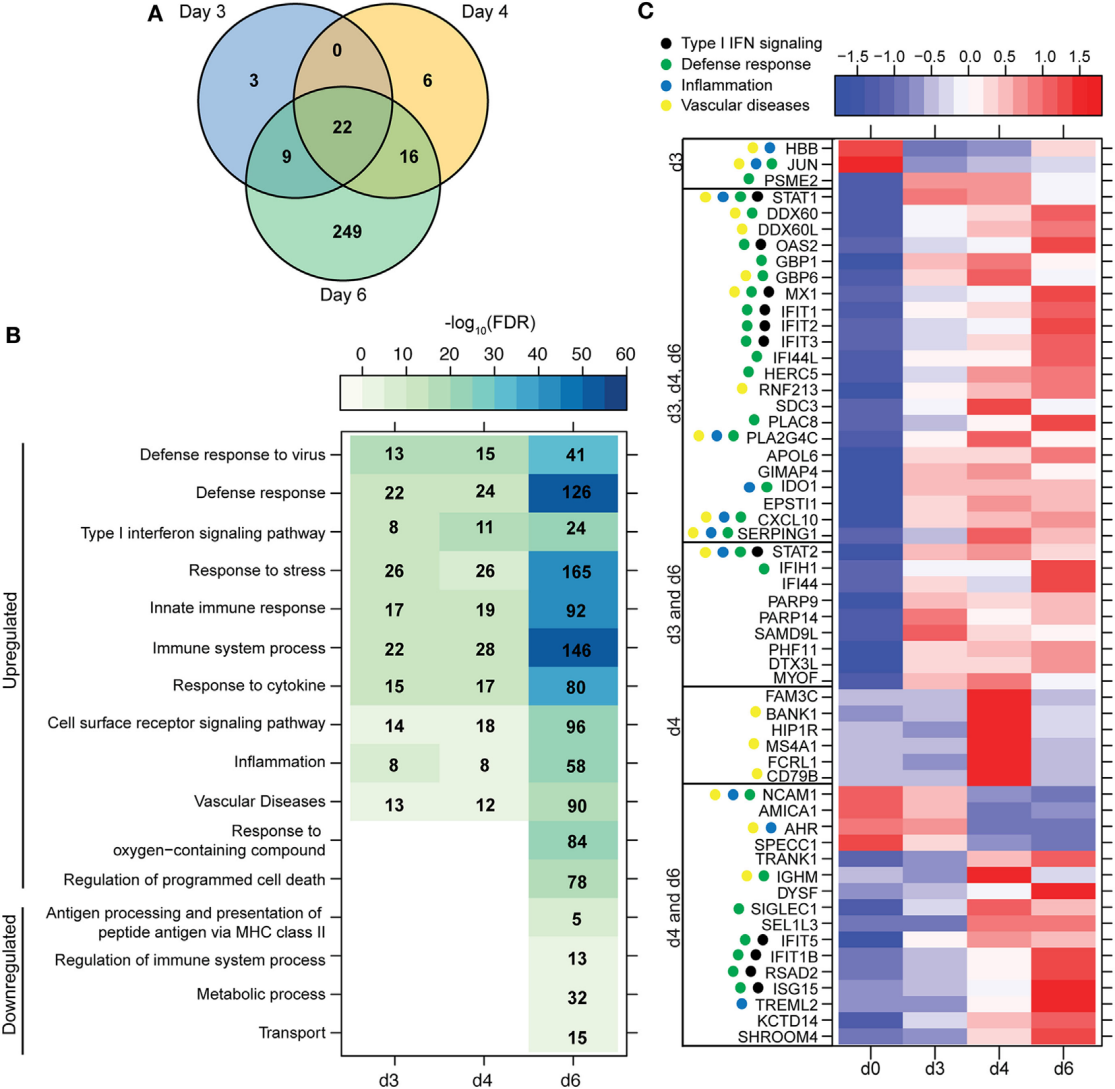
ZEBOV-Makona infection induces expression of genes involved in defense response and Type I IFN signaling in monocytes. **(A)** 3 way Venn diagram displaying overlap between differentially expressed genes (DEGs) detected 3, 4, and 6 DPI in purified monocytes. **(B)** Heatmap representing functional enrichment of DEGs 3, 4, and 6 DPI; color intensity represents the statistical significance (shown as negative log_10_ of the false discovery rate (FDR)-corrected *p*-value); range of colors is based on the lowest and highest –log_10_(FDR) values for the entire set of terms; the number of DEGs mapping to each functional enrichment term each day is listed within each box; blank boxes represent no statistical significance. **(C)** Heatmap representing gene expression (shown as absolute normalized RPKM values) of all DEGs detected 3 and 4 DPI; genes enriching to Gene Ontology or disease term are indicated by colored dot; range of colors is based on scaled and centered RPKM values of the entire set of genes (red represents increased expression while blue represents decreased expression); each column represents the median RPKM values for each DPI.

A small number of DEGs was detected 3 DPI within monocytes, which slightly increased 4 DPI, and then considerably increased 6 DPI (Figure 1A). DEGs detected 3 and 4 DPI enriched to GO terms associated with innate immunity such as type I IFN signaling pathways (Figures 2A,B). Only 2 DEGs were downregulated 3 DPI: *HBB* (Hemoglobin Subunit Beta) and transcription factor *JUN* (Jun Proto-Oncogene), which play an important role in inflammation (31) (Figure 2C). The 6 DEGs upregulated only 4 DPI included: *FAM3C* (Family With Sequence Similarity 3 Member C), which encodes Interleukin-Like EMT inducer; *HIP1R* (Huntingtin Interacting Protein 1 Related), a component of clathrin-coated vesicles; signaling adaptor *BANK1* (B-Cell Scaffold Protein with Ankyrin Repeats); and receptor *FCRL1* (FC Receptor -Like 1) (Figure 2C). Although these genes are mostly known to be expressed in B-cells, they are also expressed in activated monocytes and macrophages (32–36). Their increased expression correlated with increased frequency of CD16 + nonclassical monocytes 4 DPI (Figure S2B in Supplementary Material).

Most of the 22 DEGs that were upregulated throughout infection in monocytes (Figure 2A) were interferon-stimulated genes (ISGs) important in anti-viral defense (37), notably *GBP1* (Guanylate Binding Protein 1), *OAS2* (2′-5′-Oligoadenylate Synthetase 2), *MX1* (MX Dynamin-Like GTPase 1), and *IFIT1–3* (Interferon-Induced Protein with Tetratricopeptide Repeats 1–3) (Figure 2C). Also upregulated, were *STAT1* and *2* (Signal Transducer and Activator of Transcription 1 and 2), which serve as transcriptional activators of ISGs (37). Other DEGs of note that were upregulated throughout infection include the monocyte chemoattractant *CXCL10* (C-X-C Motif Chemokine Ligand 10) and the C1 inhibitor *SERPING1* (Serpin Family G Member 1). Additional ISGs such as *IFIT5, IFIT1B*, and *ISG15* [Interferon, Alpha-Inducible Protein (Clone IFI-15K)] as well as genes involved in receptor-mediated endocytosis such as *SIGLEC1* (Sialic Acid Binding Ig-Like Lectin 1) (38) were upregulated 4 and 6 DPI. In contrast, expression of adhesion molecules *NCAM1* (Neural Cell Adhesion Molecule 1), *AMICA* (Adhesion Molecule Interacting with *CXADR* Antigen 1), and *SPECC1* (Sperm Antigen with Calponin Homology and Coiled-Coil Domains) and transcription factor *AHR* (Aryl Hydrocarbon Receptor) were downregulated 4–6 DPI (Figure 2C).

**Figure 3:**
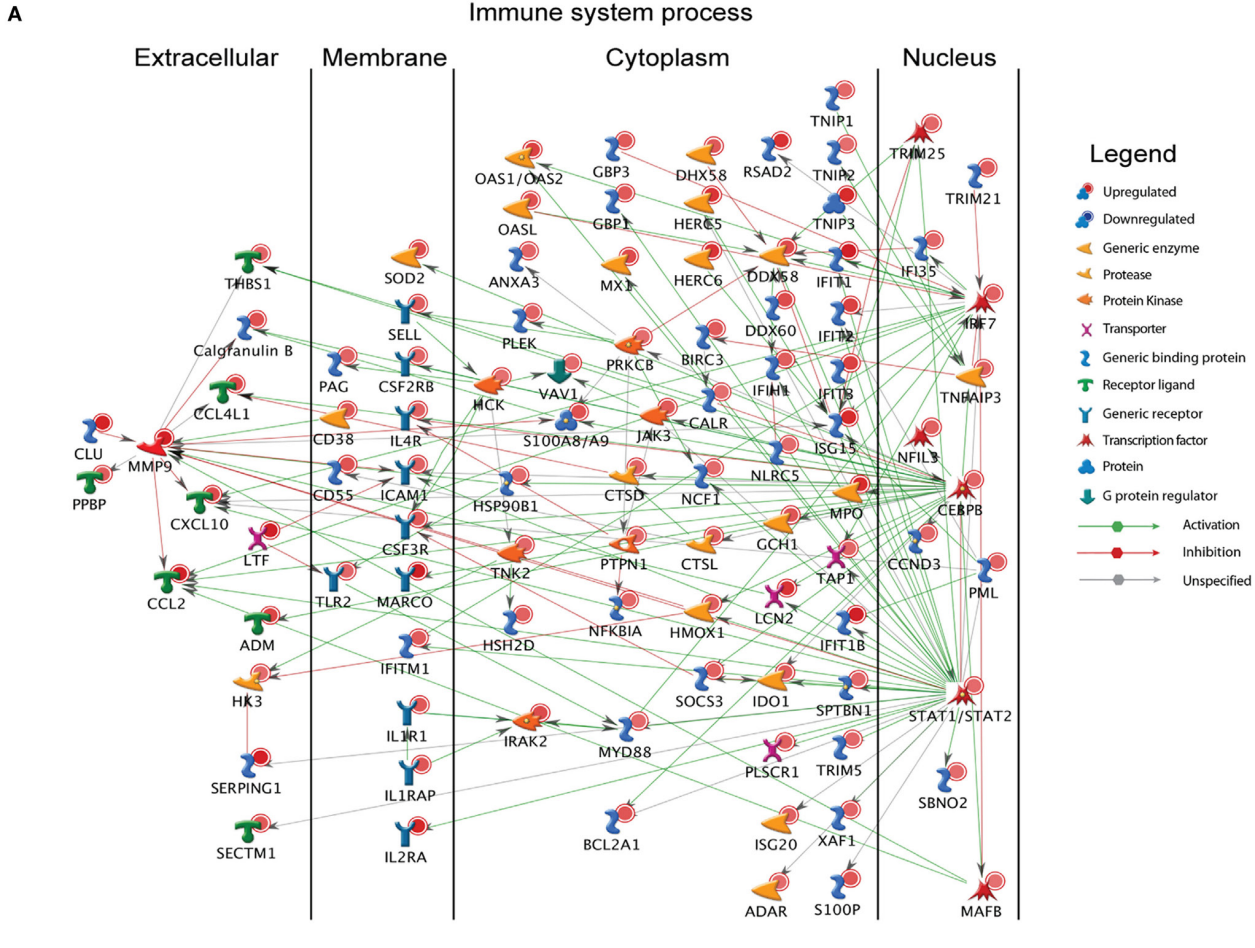

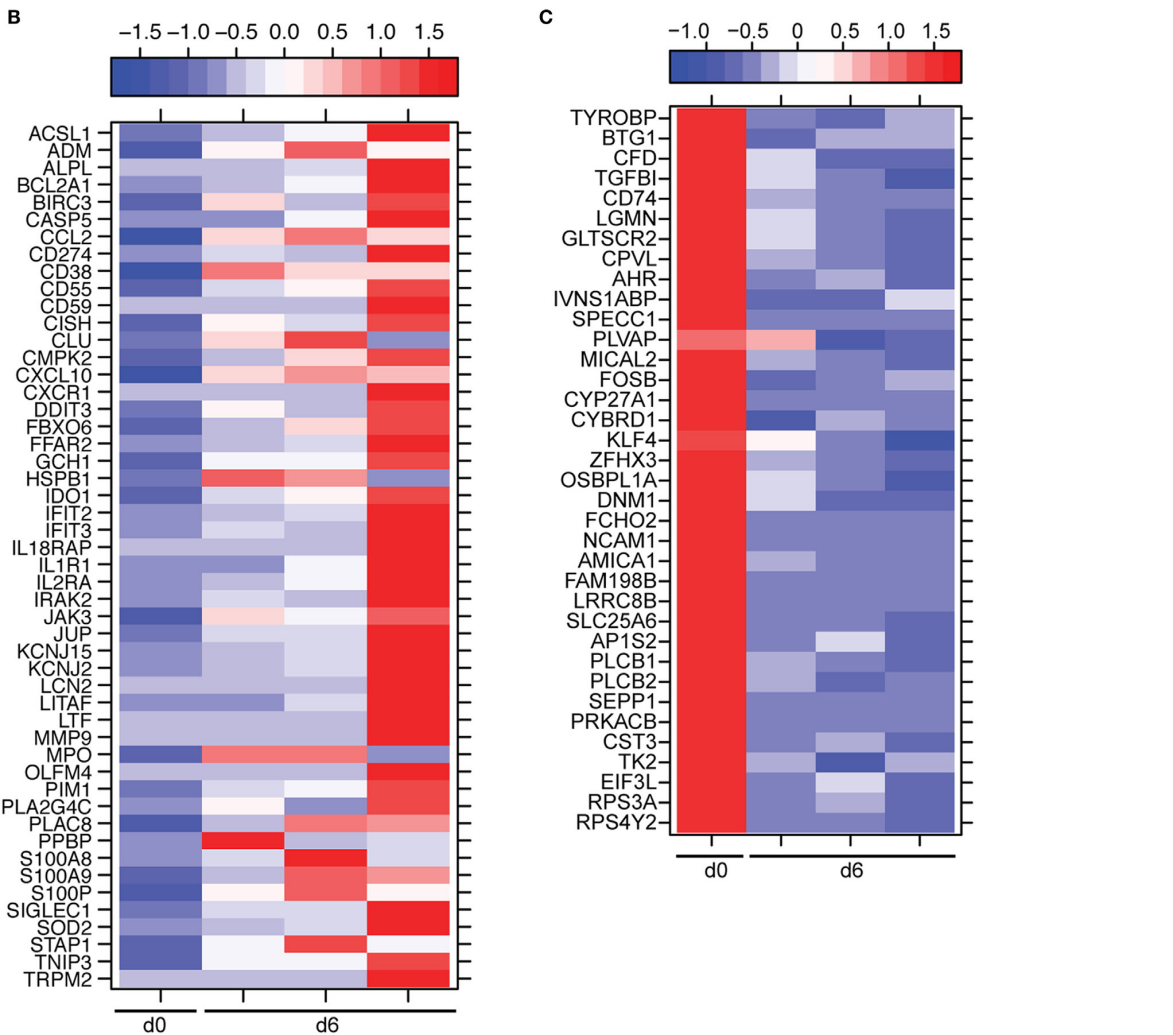
Monocytes contribute significantly to ZEBOV-associated inflammation. **(A)** Network showing direct interactions between differentially expressed genes (DEGs) upregulated 6 DPI in monocytes that enriched to “Immune system process.” **(B)** Heatmap representing gene expression (shown as absolute normalized RPKM values) of DEGs upregulated 6 DPI that enriched to “Regulation of programmed cell death” and “Response to oxygen containing compound” with a fold change ≥10.5; range of colors is based on scaled and centered RPKM values of the entire set of genes (red represents increased expression while blue represents decreased expression); day 0 is represented by the median RPKM value, while each column represents 1 animal for 6 DPI. **(C)** Heatmap representing gene expression (shown as absolute normalized RPKM values) of all downregulated genes 6 DPI; range of colors is based on scaled and centered RPKM values of the entire set of genes (red represents increased expression while blue represents decreased expression); day 0 is represented by the median RPKM value, while each column represents 1 animal for 6 DPI.

A large number of DEGs were identified 6 DPI (Table S2 in Supplementary Material). Upregulated DEGs enriched to GO terms related to defense response/inflammation, oxidative stress, and apoptosis (Figure 2B). Of the 146 DEGs that enriched to GO term “Immune system process,” 98 directly interacted with each other (Figure 3A). Expression of these genes was regulated by *STAT1, STAT2, IRF7* (Interferon Regulatory Factor), and *CEBPB* (CCAAT/Enhancer Binding Protein, Beta). Downstream mediators of these transcription factors are involved in: leukocyte recruitment, e.g., *CCL2* and *CCL4L1* (39, 40); signaling, e.g., *IRAK2* (Interleukin 1 Receptor Associated Kinase 2) (41) and *CSF3R* (Colony stimulating factor 3 receptor); and pathogen recognition, e.g., *TLR2* and *MYD88*. Other upregulated genes in this network play a role in: coagulation such as *THBS1* (Thrombospondin 1) (42); apoptosis such as *BCL2A1* (BCL2 Related Protein A1) (43); response to oxidative stress, e.g., *SOD2* (Superoxide dismutase 2) (44); positive regulation of nitric oxide synthesis such as *GCH1* (GTP Cyclohydrolase 1) (45); and vascular integrity, e.g., *MMP9* (matrix metalloproteinase-9) (46). Upregulated genes that also enriched to “Immune system process” but not included in the network include: *CD274* (PD-L1), which inhibits T-cell activation (47); neutrophil chemoattractant *IL8* (48); and monocyte chemoattractant *CCL8* (Monocyte chemotactic protein 2) (49). Notable upregulated genes that play a role in apoptosis and oxidative stress include *DDIT3* (DNA Damage Inducible Transcript 3) (50), *HSPB1* (Heat shock protein Family B Member 1) (51) *CASP5* (Caspase 5) (52), and *BIRC3* (Baculoviral IAP Repeat Containing 3) (53) (Figure 3B).

The 36 genes downregulated 6 DPI enriched to metabolic and immune processes (Figure 2B). Some of these DEGs that enriched to “Antigen processing and presentation of peptide antigen via MHC class II” include *AP1S2* (Adaptor Related Protein Complex 1 Sigma 2 Subunit), *DNM1* (Dynamin 1), *OSBPL1A* (Oxysterol Binding Protein Like 1A), *CD74* (Major histocompatibility Complex, Class II), and *LGMN* (Legumain) (Figure 3C). We also observed downregulation of transcription factors *AHR* (Aryl Hydrocarbon Receptor), *FOSB* (FBJ Murine Osteosarcoma Viral Oncogene), and *KLF4* (Kruppel-Like Factor). Finally, genes involved in translation, i.e., *EIF3L* (Eukaryotic Translation Initiation Factor 3 Subunit L), *RPS3A*, and *RPS4Y2* (Ribosomal Proteins S3 and S4 Y-Linked 2) were also downregulated (Figure 3C).

To investigate the association between transcriptional changes detected within monocytes and systemic inflammation, we performed a Spearman correlation between plasma levels of cytokines and chemokines and RPKM values of DEGs detected in monocytes 6 DPI. We observed strong correlations (*R* ≥ 0.8) between DEGs detected within monocytes 6 DPI and circulating inflammatory cytokines IL1β (42 DEGs), IL6 (53 DEGs), IFNα (92 DEGs), IL7 (40 DEGs), IFNγ (40 DEGs), IL18 (74 DEGs), and IL4 (45 DEGs) as well as chemokines MIP1α (110 DEGs), and MCP1 (45 DEGs) (Figure S4 in Supplementary Material).

### ZEBOV-Makona Infection Induces Limited Differential Gene Expression in Lymphocytes

**Figure 4:**
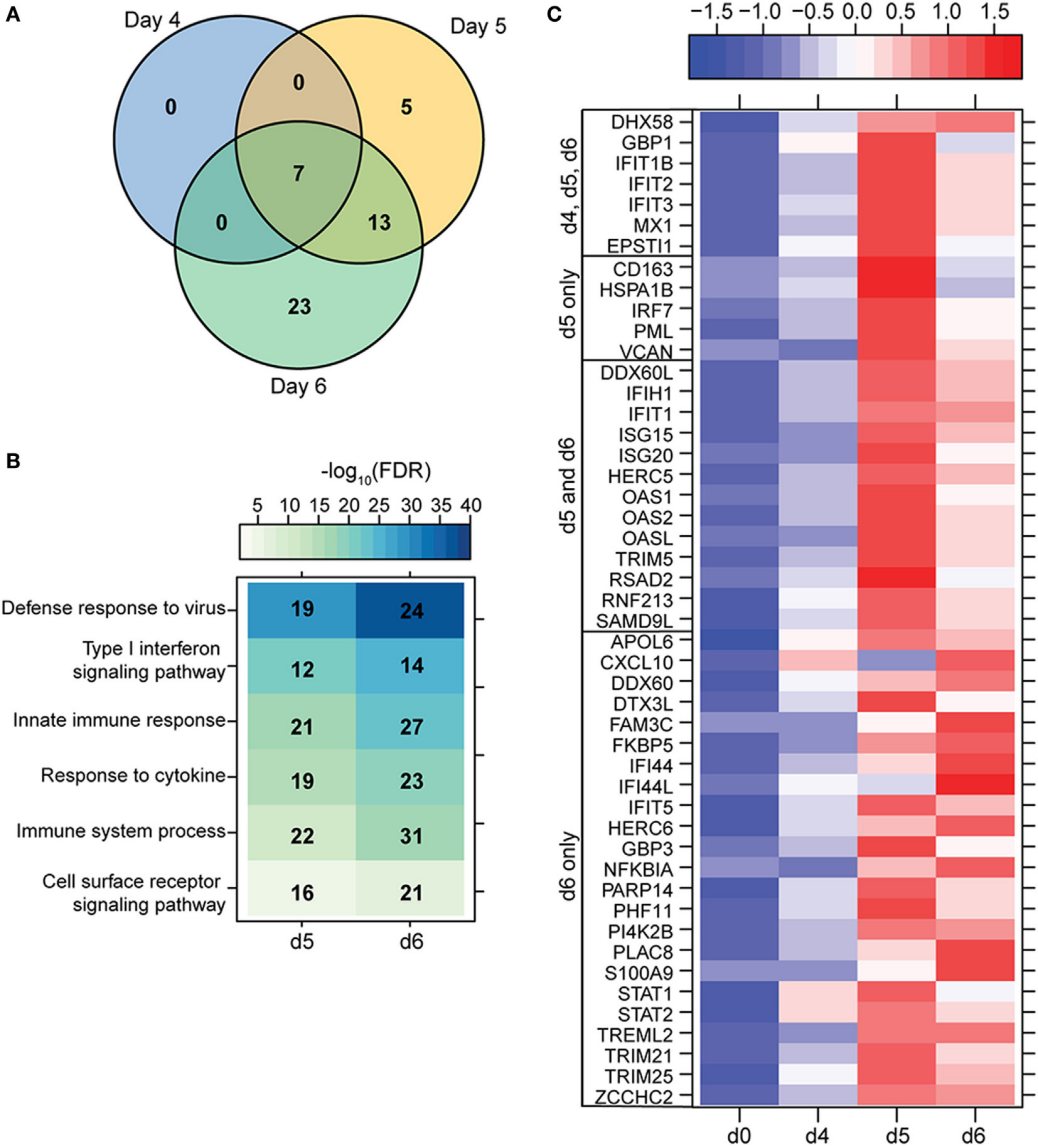
ZEBOV-Makona infection induces limited differential gene expression in T-cells. **(A)** 3 way Venn diagram displaying overlap between differentially expressed genes (DEGs) detected 4, 5, and 6 DPI. **(B)** Heatmap representing functional enrichment of DEGs 5 and 6 DPI; color intensity represents the statistical significance (shown as negative log_10_ of the false discovery rate (FDR)-corrected *p*-value); range of colors is based on the lowest and highest –log_10_(FDR) values for the entire set of terms; the number of DEGs mapping to each Gene Ontology term each day is listed within each box. **(C)** Heatmap representing gene expression (shown as absolute normalized RPKM values) of all DEGs detected 4, 5, and 6 DPI; range of colors is based on scaled and centered RPKM values of the entire set of genes (red represents increased expression while blue represents decreased expression); each column represents the median RPKM values for each DPI.

**Figure 5:**
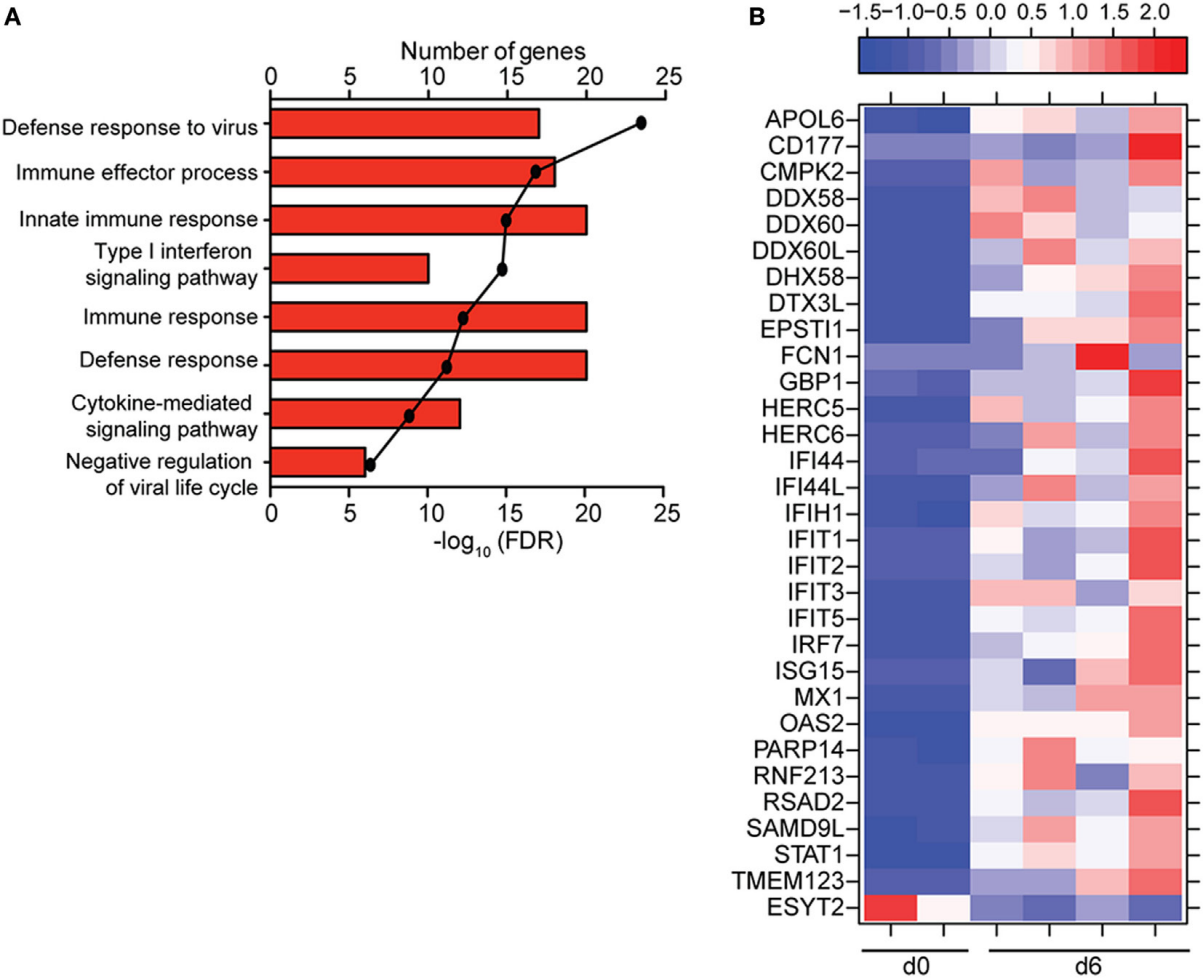
ZEBOV-Makona infection induces limited differential gene expression in B-cells. **(A)** Functional enrichment of upregulated genes detected 6 DPI; horizontal bar graphs represent number of genes that mapped to each Gene Ontology term listed while line graph represents false discovery rate (FDR)-corrected *p*-value. **(B)** Heatmap representing gene expression (shown as absolute normalized RPKM values) of all differentially expressed genes 6 DPI; range of colors is based on scaled and centered RPKM values of the entire set of genes (red represents increased expression while blue represents decreased expression); each column represents 1 animal.

To understand the consequence of ZEBOV-Makona infection on the adaptive immune response, we next determined transcriptional changes within T- and B-cells following challenge. The average purity of T-cells and B-cells was >80% and >65%, respectively (Table S1 in Supplementary Material). In contrast to monocytes, limited host and viral transcriptional changes were detected in T-cells (Figures 1A and 4A). In addition, DEGs detected in T-cells were primarily involved in “Type I IFN signaling” (Figure 4B) and consisted of ISGs that play a role in anti-viral immunity, i.e., *DHX58, GBP1, IFIT1/2, OAS1–2*, and *MX1* (Figure 4C). Last, *PHF11* (PHD Finger Protein 11) and *TREML2* (Triggering Receptor Expressed on Myeloid Cells Like 2), both of which are involved in T-cell activation (54, 55), were also upregulated 6 DPI. Similarly, we detected a limited number of gene expression changes (31 DEGs) in B-cells 6 DPI (Figure 1A), which also enriched to GO terms associated with innate immune defense to viral infection (Figure 5A) and consisted primarily of ISGs, i.e., *DHX58, GBP1, IFIT1–3, 5* (Figure 5B). Analysis of viral transcripts revealed that ZEBOV ORF and IGR transcripts in T- and B-cells were 2–12-fold lower compared to monocytes, with the exception of the trailing end region (Figure 1B). Euclidean distances also indicate that the viral transcript profile in T and B cells was more distant compared to that observed in PBMC (Figure S3 in Supplementary Material).

### Monocytes Contribute a Significant Proportion of Host Transcriptional Changes Detected in PBMC

**Figure 6:**
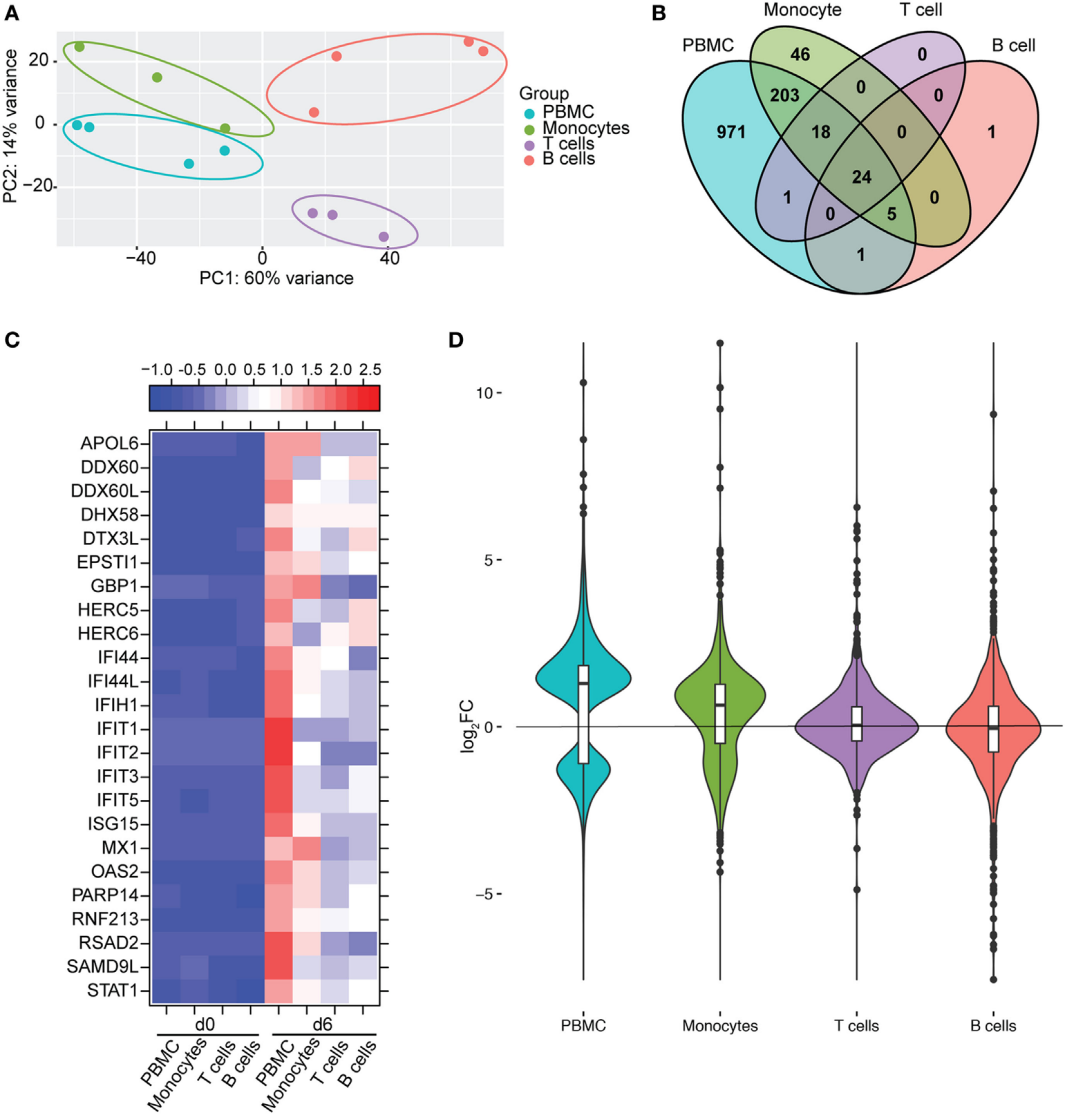
Monocytes contribute a significant proportion of transcriptional changes detected in peripheral blood mononuclear cells (PBMC). **(A)** Principal component analysis of normalized transcript numbers 6 DPI in PBMC, monocytes, T-cells, and B-cells. **(B)** 4 way Venn diagram showing overlap between differentially expressed genes (DEGs) detected 6 DPI in PBMC, monocyte, T-cells, and B-cells. **(C)** Heatmap representing gene expression (shown as absolute normalized RPKM values) of the 24 DEGs detected in PBMC as well as purified monocytes, T- and B-cells 6 DPI; range of colors is based on scaled and centered RPKM values of the entire set of genes (red represents increased expression while blue represents decreased expression); each column represents the median RPKM values on each day for each subset. **(D)** Violin plot of the log_2_FC of 971 DEGs that were exclusively detected in PBMC within each cell subset.

**Figure 7:**
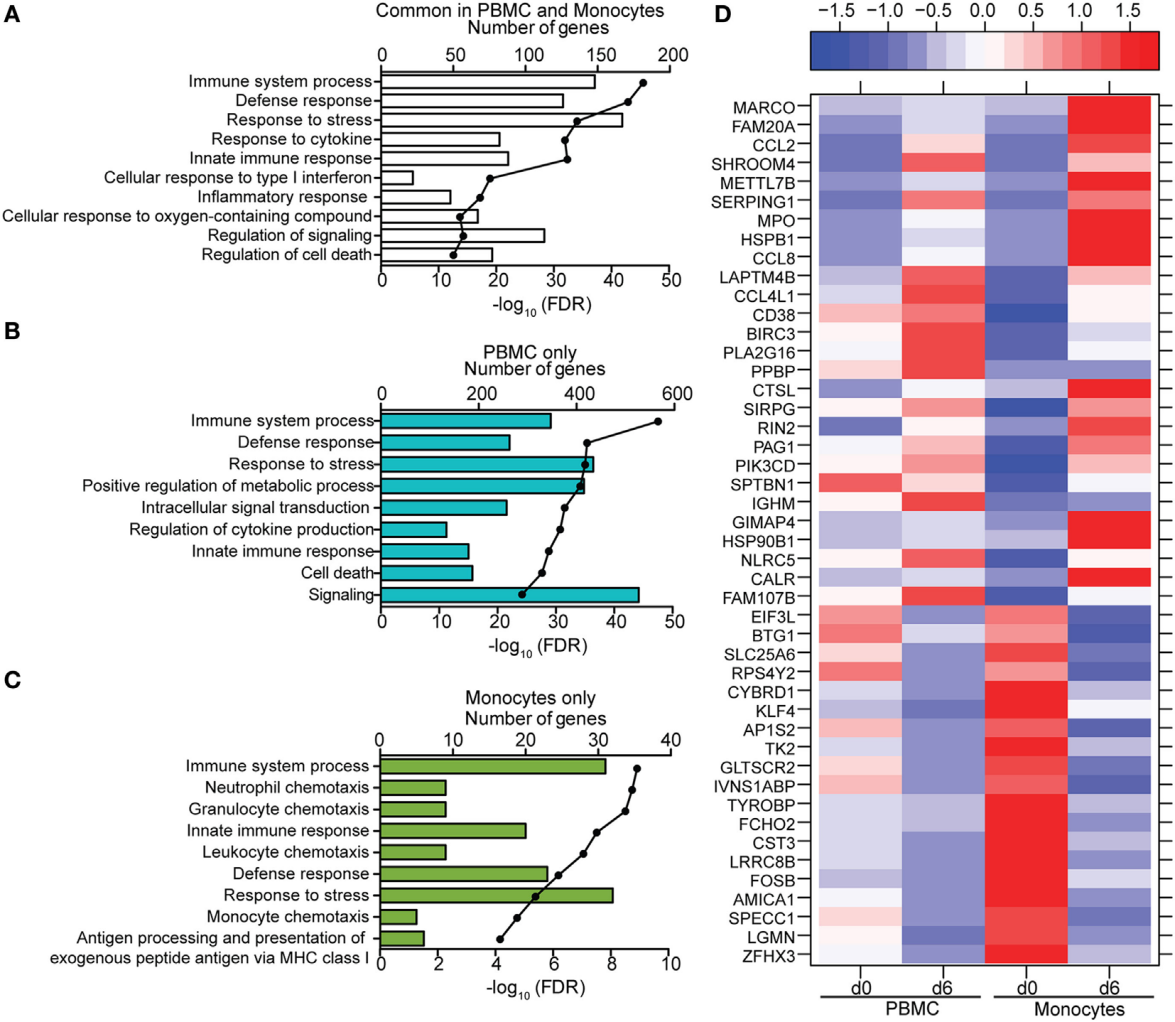
Comparison of transcriptional profiles between peripheral blood mononuclear cells (PBMC) and monocytes 6 DPI. **(A–C)** Functional enrichment of differentially expressed genes (DEGs) detected in both monocytes and PBMC **(A)**, PBMC only **(B)**, or monocytes only **(C)**; horizontal bar graphs represent number of genes that mapped to each Gene Ontology term listed while line graph represents false discovery rate (FDR)-corrected *p*-value. **(D)** Heatmap representing gene expression (shown as absolute normalized RPKM values) of the 46 DEGs that were only detected in monocytes 6 DPI; corresponding RPKM values in PBMC are shown for comparison; range of colors is based on scaled and centered RPKM values of the entire set of genes (red represents increased expression while blue represents decreased expression); each column represents the median RPKM values on each day for each subset.

We next compared gene expression profiles of PBMC, monocytes, T-cells, and B-cells 6 DPI, the time point at which Makona-ZEBOV infection caused the most significant clinical signs and transcriptional changes [Table 1 (26)]. Principle component analysis of normalized reads across all groups showed that monocytes were transcriptionally more similar to PBMC than T-cells and B-cells (Figure 6A). Comparison of all protein coding DEGs among the four groups revealed that 20% of DEGs detected in PBMC were also differentially expressed within monocytes. In contrast, the DEGs detected in T-cells and B-cells accounted for only 3.4 and 2.5% of the DEGs detected in PBMC (Figure 6B). As expected, the 24 DEGs common to all 4 groups play a role in Type I IFN response (Figure 6C). The 971 genes that were only differentially expressed in PBMC had lower fold changes and their expression did not reach the statistical cutoff of FDR adjusted *p*-value ≤ 0.05 and average RPKM ≥ 5 in each individual cell subset (Figure 6D). The 250 DEGs detected in both monocytes and PBMC enriched to GO terms linked to immunity and cell death (Figure 7A). Functional enrichment of the 971 genes exclusive to PBMC are involved in similar biological processes as the 250 DEGs found in monocytes related to host defense, innate immune response, and inflammation (Figure 7B). These data suggest that these DEGs are only significant when transcriptional changes across all subsets are included in the analysis. The 46 DEGs detected exclusively in monocytes 6 DPI mapped to GO terms such as “Innate immune response” and “Antigen processing and presentation of exogenous peptide antigen” and had higher magnitude of expression compared to PBMC, suggesting that their expression was diluted by the inclusion of the additional subsets in PBMC analysis (Figures 7C,D).

## Discussion

In this manuscript, we report the first *in vivo* longitudinal analysis of viral and host transcriptional changes in purified monocytes, T-cells, and B-cells isolated from cynomolgus macaques following ZEBOV-Makona infection. The susceptibility of monocytes to ZEBOV infection has been controversial. Some *in vitro* studies showed that filoviruses (MARV, ZEBOV) can replicate in both primary monocytes and MDMs with similar virus growth kinetics (6, 7, 15), whereas other *in vitro* studies reported delayed ZEBOV entry in undifferentiated monocytes compared to macrophages (17). Similarly, PMA-naïve THP-1 cells were resistant to virus like particles pseudotyped with ZEBOV GP and become permissive only following differentiation (17). Our data strongly suggest circulating blood monocytes are permissive to ZEBOV replication *in vivo* given the significant increase in ZEBOV reads 6 DPI. Furthermore, as previously reported in Vero E6 cells, NP was one of the most transcribed genes, whereas L was the least transcribed (56). These observations are in line with earlier studies reporting viral antigens within DC and monocytes in draining lymph nodes following intramuscular challenge with EBOV (4). In support of the ability of monocytes to permit viral replication, genes involved in receptor-mediated endocytosis 4 DPI (*SIGLEC1* and *HIP1R*) as well as Cathepsin L, which plays a role in ZEBOV-GP mediated entry (57), were upregulated 6 DPI.

Differentially expressed genes were detected as early as 3 DPI in monocytes, confirming they are among the early targets of ZEBOV infection (4). Although we did not detect increased transcripts of IFNα/β within monocytes, we saw increased expression of ISGs (e.g., *IFIT1–4, OAS1–2, OASL, RSAD2, ISG15*, and *GBP1*). Similarly, ISGs (*IFIT1–2, GBP1, OASL*) have been detected in MDMs 6 h post *in vitro* infection with ZEBOV-Mayinga (EBOV strain isolated in 1976) in the absence of increased IFNα/β expression (58). A possible reason for elevated ISGs in our study is that monocytes may be responding to low levels of IFNα/β secreted from non-infected DCs and monocytes that are activated by viral debris, such as “shed” GP (59). Moreover, it has been previously shown that ZEBOV virion binding mediated by GP is responsible for early macrophage transcriptional responses before the detection of viral replication (58). A second possible explanation is that the increased expression of ISGs is independent of the activation of canonical Type I IFN signaling. Pulit-Penaloza et al. reported the upregulation of *OAS1a, OAS1b, IRF1*, and *IRF7* in STAT1^−/−^, STAT2^−/−^, and IFNα/β receptor^−/−^ mouse embryonic fibroblasts infected with West Nile Virus (60), indicating that an alternative activation mechanism exists for inducing ISG expression in the absence of Type I IFN signaling.

We observed the most transcriptional changes in ZEBOV-Makona infected monocytes 6 DPI. A large number of genes upregulated 6 DPI are important for inflammatory responses such as *CXCL10, SERPING1, PPBP, IL8, CSF3R, IL1R1, MYD88, IRAK2*, and *NFKBIA*. The increased expression of chemokines *CCL2, CCL4L1*, and *CCL8* may be important for the recruitment of additional monocytes and DCs to sites of infection allowing ZEBOV to disseminate. Furthermore, additional analysis revealed a strong association between DEGs detected in monocytes 6 DPI to circulating levels of inflammatory immune mediators including IFNα, IL-6, MIP-1α, and MCP-1 (CCL2). Overall, there was strong agreement between the type of association (positive or negative) with immune mediators and observed changes in gene expression. For instance, expression of most ISGs were positively associated with IFNα. Although some genes were predicted to be negatively regulated by IL1β and IL18, their expression increased, suggesting that they are positively regulated by other mediators not included in this analysis.

Expression of genes involved in apoptosis (*OLFM4, BCL2A1, CASP5*, and *BIRC3*) and unfolded protein response (*DDIT3, FBX06, HSPB1*, and *HSPA5*) were significantly increased. Interestingly, HSPB1 and HSPA5 proteins have been identified as EBOV associated host factors, and targeting of HSPA5 *in vivo* using PMOs in mice before challenge increased survival rates (61, 62). Additionally, we identified an increase in transcripts associated with the production of reactive nitric/oxygen species (*GCH1* and *SOD2)* and vascular function (*ADM, MMP9, THBS1*, and *SERPINA1*). This is consistent with documented reports of increased nitric oxide in plasma of fatal cases compared to patients who survived (10). Furthermore, increased expression of *MMP9*, as well as other metalloproteinase genes, in the spleen of mice challenged with mouse adapted (MA)-ZEBOV have been associated with lethality (63). Interestingly, we did not detect changes in tissue factor (TF) transcripts, which has previously been reported to increase in lymphoid macrophages collected from EBOV challenged NHPs (16). However, TF is produced by tissue macrophages, therefore transcriptional analysis of circulating monocytes may have precluded us from observing changes in TF. In line with a recent study that revealed increased numbers of T-cells expressing inhibitory molecule PD-1 in ZEBOV-Makona fatal cases (64), we detected an upregulation of *CD274* (*PD-L1*), which inhibits TCR-mediated activation. Although it did not reach our statistical cutoff, we did detect an increase in PD-1 transcripts (fold change of 3.29) 6 DPI in T-cells. In contrast, genes associated with antigen processing and presentation (*AP1S2, OBPL1, CD74, LGMN*) were downregulated. Collectively, these data suggest that infected monocytes and macrophages are likely to be one of the major drivers of excessive inflammation during ZEBOV infection and provide insight into the failure of initiating an adaptive immune response. We also detected a downregulation of genes associated with translation, including genes encoding for translation initiation factors and ribosomal proteins, a strategy that may be employed by the host to limit ZEBOV replication and spread.

Our observations differ from an *in vitro* study by Wahl-Jensen et al. where they identified 88 DEGs in macrophages within the first 6 h after ZEBOV-Mayinga infection (58). The earlier detection of DEGs in the Wahl-Jensen study correlates with the faster onset of clinical symptoms seen after Mayinga infection compared to Makona (27). Additionally, the earlier detection of DEGs could be due to the high multiplicity of infection used *in vitro* that may not be reached until later time points during *in vivo* infection. Of the 88 DEGs identified in the Wahl-Jensen study, 20 were common with our data set including genes involved in: apoptosis (*TNFAIP3, BIRC3*); immune defense, inflammation, and chemotaxis (*TNFAIP6, TX3, IL8, CCL8, PPBP, TRIM25*, and *IL15RA*); adhesion (*ICAM1*); oxidative stress (*SOD2, GCH1*); and ISGs (*IFIT1–2, OASL, GBP1*). In line with our findings, a recent study by Liu et al. showed that patients who succumbed to ZEBOV-Makona infection had a stronger upregulation of acute phase responses and an increased abundance of ISG transcripts compared to survivors (65). Moreover, longitudinal transcriptomic analysis in peripheral blood from a surviving male health care worker exposed to EBOV during the 2013–2016 ZEBOV-Makona epidemic revealed that transcripts that positively correlated with viremia enriched to GO terms associated with activation of antiviral responses (including Type I IFN signaling), inflammation, oxidative stress, DNA damage and cell death (66). Additionally, transcripts with a positive correlation with D-dimer levels and negative correlation with platelet levels functionally enriched to processes associated with macrophage and neutrophil recruitment, macrophage death, antiviral response, and phagocytosis (66).

Additional bioinformatics analysis revealed that 62% of DEGs detected within monocytes 6 DPI were shared with the transcriptional changes in MDMs and macrophages differentiated from human induced pluripotent stem cells (IPSDMs) following 6 h LPS stimulation (Figure S5A in Supplementary Material) (29). These common genes enriched to GO terms associated with inflammation, response to cytokine, and response to Type I interferon (Figure S5B in Supplementary Material). This observation suggests that ZEBOV infection may result in monocyte activation. In contrast, the 40% of DEGs that were unique in our data set enriched to wounding, coagulation, hemostasis, and response to oxidative stress, which is in line with the development of EVD (Figure S5C in Supplementary Material).

In contrast to monocytes, our data indicate that T- and B-cells are less permissible to ZEBOV replication, as evidenced by the lower number of viral transcripts in lymphocytes. Additionally, the abundance of viral transcripts was comparable among all ZEBOV ORFs, suggesting abortive transcription. This is consistent with previous *in vivo* and *in vitro* reports that show ZEBOV does not replicate within lymphocytes and that lymphocyte apoptosis is likely an indirect result of ZEBOV induced upregulation of negative regulators and inflammatory mediators (15, 23, 24). The low number of ZEBOV reads detected in T- and B-cells 6 DPI may be due to increased amounts of circulating fibrin bound to cells and viral particles resulting in viral genome carry-over during cell separation.

The small number of DEGs detected in T and B cells 6 DPI were overwhelmingly ISGs. This increase in ISG expression within lymphocytes correlates with the large increase in circulating IFNα levels observed 6DPI. However, fold changes of ISG expression were much lower in lymphocytes compared to monocytes. Previous experiments have demonstrated that IFNα subtypes can activate the JAK/STAT pathway and induce ISGs in T-cells albeit at a lower magnitude compared to DCs (67). Interestingly, we did not detect changes in the expression of genes that play a role in lymphocyte apoptosis such as *FAS, caspase*, and *TRAIL* despite the lymphopenia we observed *in vivo*. In contrast, our previous analysis of transcriptional changes within blood samples isolated from the same animals showed a large decrease in expression of lymphocyte related genes and a robust increase in expression of pro-apoptotic genes (26). These observations strongly suggest that the absence of such transcripts in this study is due to the fact that T- and B-cells purified from PBMC, are mostly viable cells not undergoing cell death. Importantly, we were unable to detect any signatures of an adaptive immune response, such as antiviral cytokine IFNγ or cytolytic molecules perforin and granzyme B, needed to clear viral infection and establish memory. A recent study showed production of immunosuppressive mediators IL-10 and PD-L1 is dependent on Type I IFN signaling in a mouse model of lymphocytic choriomeningitis virus infection (68, 69). Therefore, continuous Type I IFN response, including sustained expression of ISGs, driven by ZEBOV-Makona infection may inhibit the ability of lymphocytes to develop into an effective defense response. One limitation in our study was the low purity of B cells isolated at early time points post-infection. However, the transcriptional profile within T cells, which had very little contamination, was similar to B cells, suggesting any contamination had little influence on the gene expression changes we observed. Another limitation is that we were not able to determine transcriptional changes within DCs due to very low cell recovery following sequential isolation using magnetic beads given that this subset make up less than 1% of the PBMC population and was further decreased 6 DPI (26).

In line with the larger transcriptional changes observed, DEGs in monocytes contributed a more significant portion of transcriptional changes (~20% compared to 2–3%) detected in PBMC 6 days post ZEBOV infection compared to T- and B-cells. The 250 common DEGs detected in the purified monocyte fraction and PBMC play a central role in immunity, inflammation, and cell death. However, 971 DEGs that were detected within total PBMC did not meet the criteria for DEGs within purified monocytes, T-, or B-cells. These 971 DEGs enriched to biological processes involved in innate as well as adaptive immunity. Therefore, it is possible that expression changes in these genes are only significant when combining changes in all three cell subsets. Conversely, by enriching for monocytes, changes in expression of genes important for chemotaxis and antigen presentation became more pronounced, likely because the transcription profile was more specific to these cells rather than diluted by the global PBMC transcriptional profile.

In summary, our data revealed that monocytes, but not lymphocytes, are supportive of ZEBOV replication. Our findings also indicate that while ZEBOV replication in monocytes may suppress IFNα/β mRNA expression (7, 70), it does not suppress ISG induction in infected cells. The gene expression patterns detected identify monocytes as one of the key players in ZEBOV pathogenesis by initiating events that contribute to characteristic symptoms of EVD including inflammation, vascular permeability, coagulation defects, and production of reactive oxygen species.

## Ethics Statement

This study, including all protocols, was approved by the University of Texas Medical Branch at Galveston Institutional Animal Care and Use Committee in accordance with state and federal statutes and regulations relating to experiments involving animals and the Institutional Biosafety Committee.

## Author Contributions

IM and TG conceived and designed experiments. AM, KV, CW, CM, JG, RC, KA, and AJ conducted the experiments. All authors contributed to data analysis and interpretation, manuscript preparation, and final approval.

## Conflict of Interest Statement

The authors declare that the research was conducted in the absence of any commercial or financial relationships that could be construed as a potential conflict of interest.
