# Supplementary material for: Transcriptome Analysis of Circulating Immune Cell Subsets Highlight the Role of Monocytes in Zaire Ebola Virus Makona Pathogenesis
Source: Front Immunol. 2017 Oct 26;8:1372. doi: 10.3389/fimmu.2017.01372 (PMC5662559; doi:10.3389/fimmu.2017.01372)
Supplement: Supplementary file 1 [file table_1.docx]

**Table S1**. Average cell subset purity (%) per day

| **Days post infection** | **d0** | **d1** | **d2** | **d3** | **d4** | **d5** | **d6** |
| --- | --- | --- | --- | --- | --- | --- | --- |
| **Monocytes** | **70.5** | **80.6** | **88.5** | **73.9** | **77.9** | **NA** | **73.5** |
| T cells | 11.76 | 8.43 | 1.67 | 18.15 | 6.93 | NA | 3.19 |
| B cells | 5.89 | 4.17 | 4.81 | 2.81 | 7.13 | NA | 9.36 |
| Dendritic Cells | 6.30 | 5.70 | 5.06 | 4.42 | 4.04 | 6.47 | 6.92 |
| **T cells** | **87.6** | **89.7** | **85.6** | **89.9** | **85.1** | **71.8** | **82.1** |
| Monocytes | 1.56 | 0.63 | 0.89 | 0.43 | 1.56 | 11.57 | 4.09 |
| B cells | 3.04 | 2.78 | 4.04 | 0.5 | 2.43 | 5.84 | 6.11 |
| Dendritic Cells | 2.00 | 1.66 | 1.73 | 2.52 | 2.68 | 4.74 | 1.07 |
| **B cells** | **65.2** | **65.7** | **72.8** | **NA** | **NA** | **NA** | **77.4** |
| Monocytes | 21.34 | 18.53 | 6.65 | NA | NA | NA | 7.37 |
| T cells | 3.08 | 2.72 | 3.69 | NA | NA | NA | 4.76 |
| Dendritic Cells | 4.45 | 5.75 | 4.12 | NA | NA | NA | 2.95 |

NA: Samples not available
