# Supplementary figures and images for: Transcriptome Analysis of Circulating Immune Cell Subsets Highlight the Role of Monocytes in Zaire Ebola Virus Makona Pathogenesis
Source: Front Immunol. 2017 Oct 26;8:1372. doi: 10.3389/fimmu.2017.01372 (PMC5662559; doi:10.3389/fimmu.2017.01372)

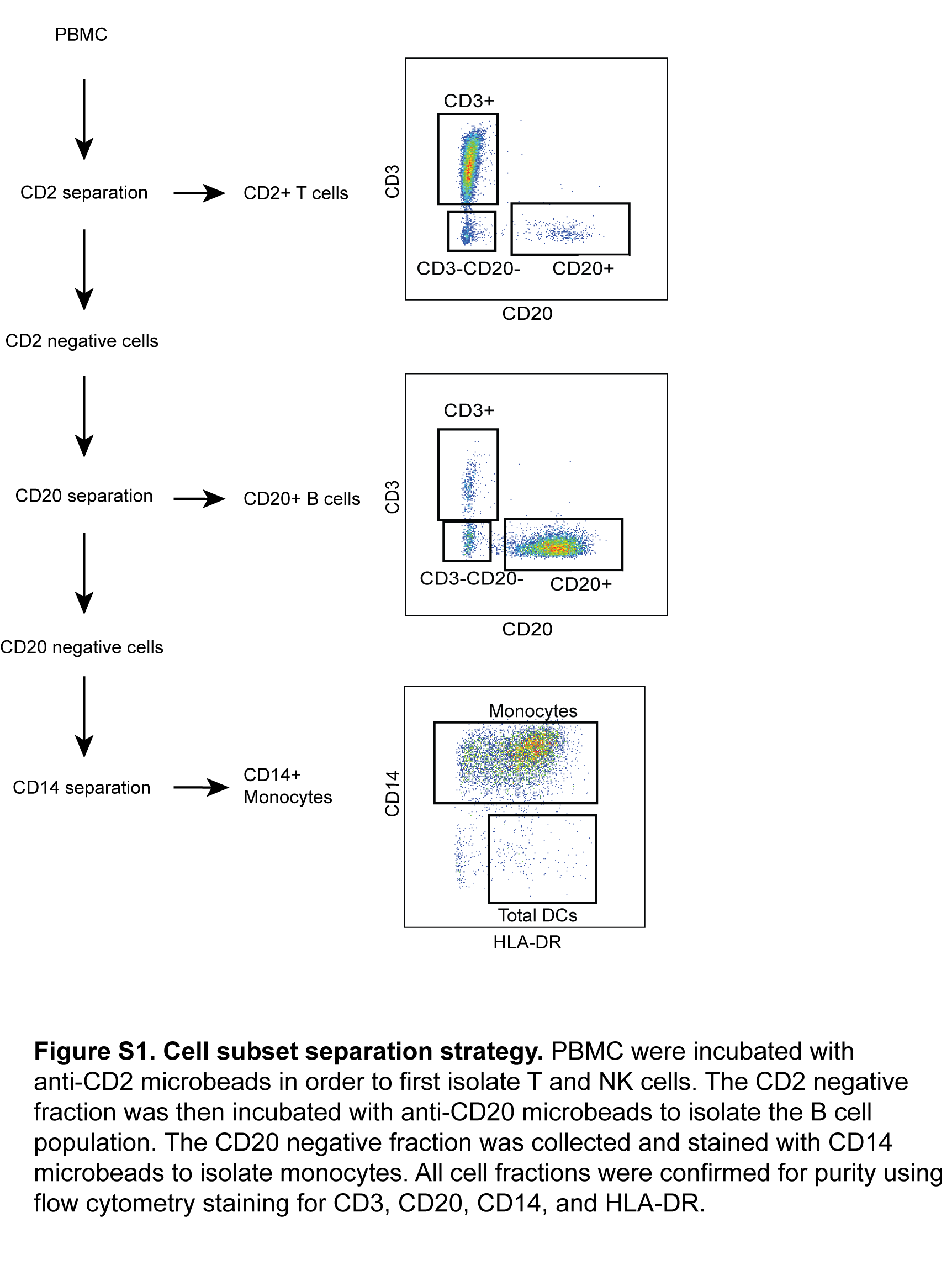

Supplement: Supplementary file 3 [file image_1.tif]

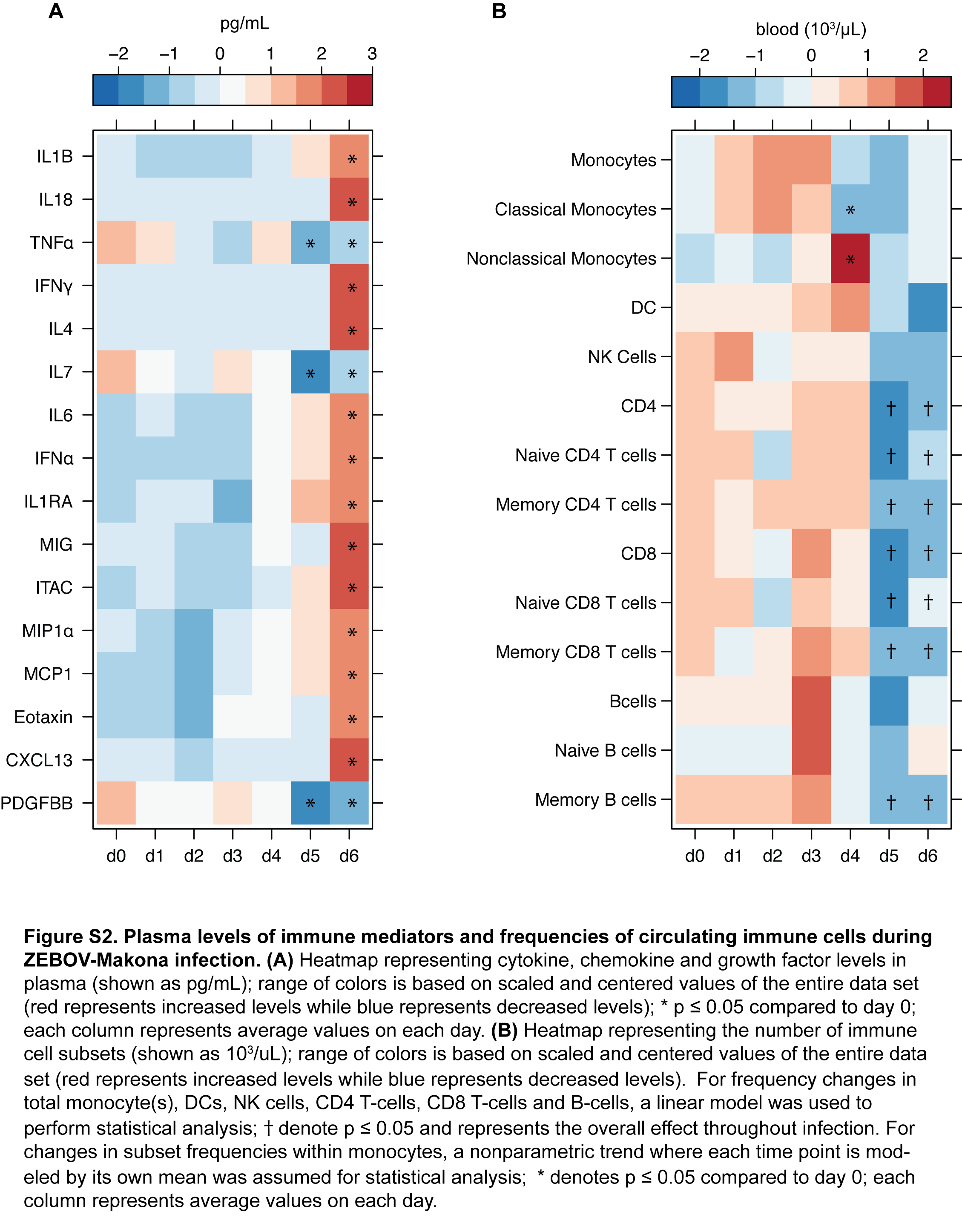

Supplement: Supplementary file 4 [file image_2.tif]

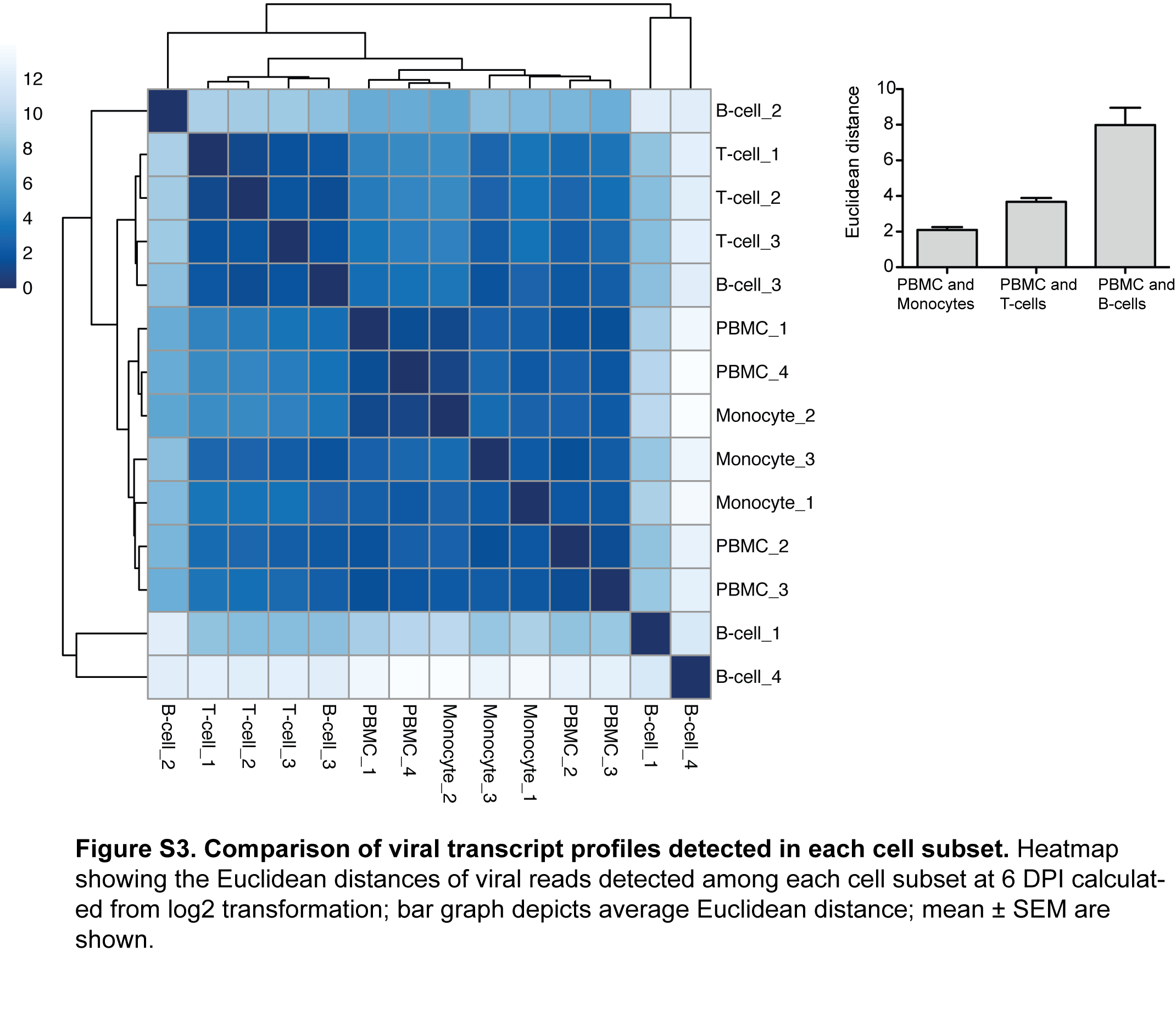

Supplement: Supplementary file 5 [file image_3.tif]

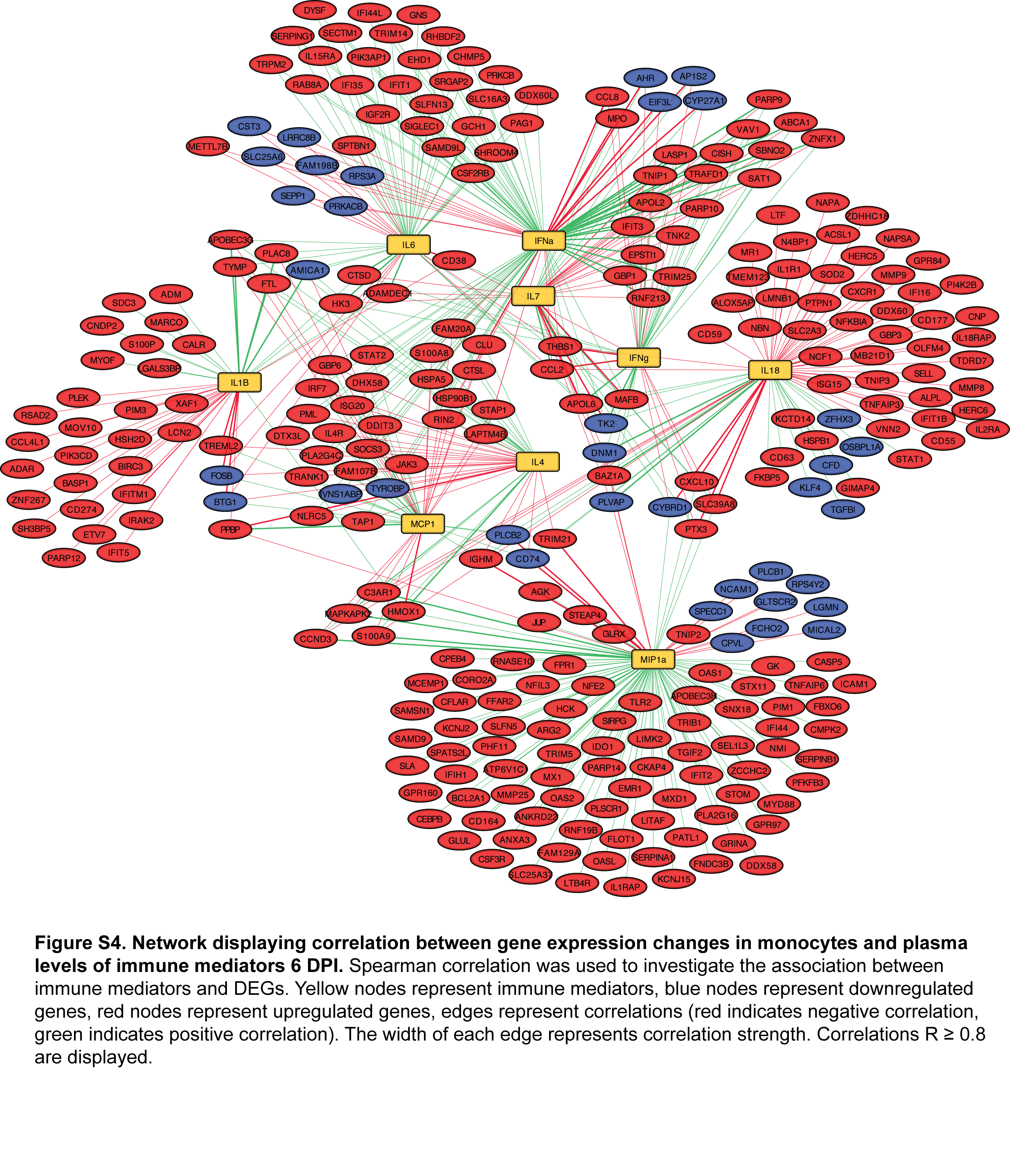

Supplement: Supplementary file 6 [file image_4.tif]

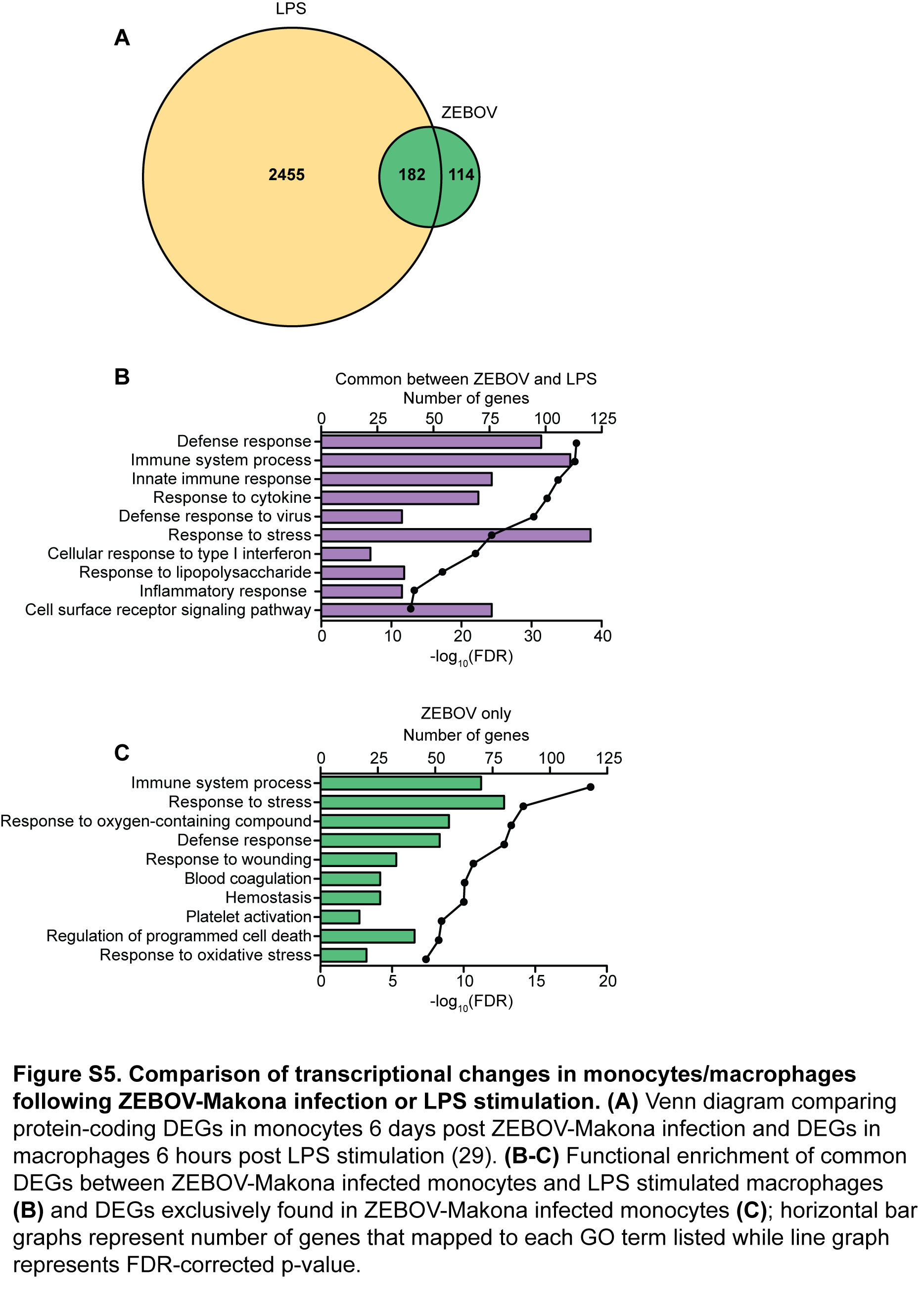

Supplement: Supplementary file 7 [file image_5.tif]
